# A Proposed Curricular Framework for an Interprofessional Approach to Deprescribing

**DOI:** 10.1007/s40670-022-01704-9

**Published:** 2023-02-23

**Authors:** Barbara Farrell, Lalitha Raman-Wilms, Cheryl A. Sadowski, Laurie Mallery, Justin Turner, Camille Gagnon, Mollie Cole, Allan Grill, Jennifer E. Isenor, Dee Mangin, Lisa M. McCarthy, Brenda Schuster, Caroline Sirois, Winnie Sun, Ross Upshur

**Affiliations:** 1 Bruyère Research Institute, 43 Bruyère St, Ottawa, ON K1N 5C8 Canada; 2 Department of Family Medicine, University of Ottawa, Ottawa, ON Canada; 3 School of Pharmacy, University of Waterloo, Waterloo, ON Canada; 4 College of Pharmacy, Rady Faculty of Health Sciences, University of Manitoba, Winnipeg, MB Canada; 5 Centre On Aging, Winnipeg, MB Canada; 6 Faculty of Pharmacy & Pharmaceutical Sciences, University of Alberta, Edmonton, AB Canada; 7 Faculty of Medicine, Dalhousie University, Halifax, NS Canada; 8 Faculty of Pharmacy, University of Montreal, Montreal, QC Canada; 9 Centre de Recherche, Institut Universitaire de Gériatrie de Montréal, Montréal, QC Canada; 10 Faculty of Pharmacy and Pharmaceutical Sciences, Monash University, Victoria, Australia; 11 Canadian Deprescribing Network, Centre de Recherche, Institut Universitaire de Gériatrie de Montréal, Montréal, QC Canada; 12 Canadian Gerontological Nursing Association, Calgary, AB Canada; 13 Dept. of Family & Community Medicine, University of Toronto, Toronto, ON Canada; 14 Markham Family Health Team, Markham, ON Canada; 15 Ontario Renal Network, Toronto, Canada; 16 College of Pharmacy, Faculty of Health and Department of Community Health and Epidemiology, Faculty of Medicine, Dalhousie University, Halifax, NS Canada; 17 David Braley & Nancy Gordon Chair in Family Medicine, Department of Family Medicine, McMaster University, Hamilton, ON Canada; 18 Department of General Practice, University of Otago, Christchurch, New Zealand; 19 Institute for Better Health and Pharmacy Department, Trillium Health Partners, Mississauga, ON Canada; 20 Leslie Dan Faculty of Pharmacy & Department of Family and Community Medicine, University of Toronto, Toronto, ON Canada; 21 Women’s College Research Institute, Toronto, ON Canada; 22 College of Medicine (Regina Campus), University of Saskatchewan, Regina, Saskachewan Canada; 23 Centre d’excellence Sur Le Vieillissement de Québec & VITAM - Research Centre On Sustainable Health, Québec, Canada; 24 Faculty of Pharmacy, Université Laval, Québec, Canada; 25 Faculty of Health Sciences, Ontario Tech University, Oshawa, ON Canada; 26 Ontario Shores Centre for Mental Health Sciences, Whitby, ON Canada; 27 Division of Clinical Public Health, Dalla Lana School of Public Health, University of Toronto, Toronto, ON Canada; 28 Bridgepoint Collaboratory for Research and Innovation, Toronto, ON Canada; 29 Lunenfeld Tanenbaum Research Institute, Sinai Health, Toronto, ON Canada; 30 Department of Family and Community Medicine, Dalla Lana School of Public Health, University of Toronto, Toronto, ON Canada

**Keywords:** Deprescribing, Polypharmacy, Education, medical, Curriculum, Competency, Geriatrics

## Abstract

Deprescribing involves reducing or stopping medications that are causing more harm than good or are no longer needed. It is an important approach to managing polypharmacy, yet healthcare professionals identify many barriers. We present a proposed pre-licensure competency framework that describes essential knowledge, teaching strategies, and assessment protocols to promote interprofessional deprescribing skills. The framework considers how to involve patients and care partners in deprescribing decisions. An action plan and example curriculum mapping exercise are included to help educators assess their curricula, and select and implement these concepts and strategies within their programs to ensure learners graduate with competencies to manage increasingly complex medication regimens as people age.

**Supplementary Information:**

The online version contains supplementary material available at 10.1007/s40670-022-01704-9.

## Background: the Rationale for a Deprescribing Competency Framework

As the population ages, healthcare professionals will encounter older adults with multiple chronic conditions taking many medications, for whom the balance of potential benefit and harm from medications may shift over time. Decisions related to continuing, reducing, or stopping medications, especially in older adults, can be difficult. There is a growing need to standardize the teaching and assessment of deprescribing knowledge and skills to address these challenges.

In 2016, one-quarter of Canadian seniors were prescribed ten or more medications, with half treated with at least one medication on the American Geriatrics Society Beers Criteria® list of “potentially inappropriate medications” (PIMs) [1, 2]. The prevalence of PIMs and polypharmacy (use of unnecessary medications, or medications where harm outweighs benefit) [3, 4] has risen steadily internationally over the past two decades [5–8]. As the number of PIMs increases, so too does the risk of adverse drug events (ADEs) [9], drug-drug interactions [10], hospital admissions [9, 11, 12], and mortality [13, 14]. Medications may become potentially inappropriate when they are continued longer than intended (e.g., proton pump inhibitors, antidepressants) [15], or when many are used to treat multiple chronic conditions resulting in additive harmful effects [16–18], or when they are continued in the context of frailty [19–21], dementia [22], or end-of-life care [23, 24] when the beneficial effect is no longer evidence-based [25, 26], apparent, or aligned with the patient’s goals of care. Growing risk of medication harm is related to progressive functional decline and pharmacodynamic changes that, for example, increase sensitivity to cardiovascular medications, anticoagulants, opioids, antipsychotics, and benzodiazepines [27–30].

Deprescribing is the patient-centered, planned, and supervised process of dose reduction or stopping of a medication that may be causing harm or may no longer be providing benefit [31]. It is a fundamental component of appropriate prescribing which can improve quality of life and medication adherence while reducing ADEs, system cost, and patient expense [32–34]. Deprescribing, with appropriate monitoring, is safe [35], with non-randomized studies suggesting deprescribing can reduce mortality [36].

There is international agreement that problematic polypharmacy must be addressed and that deprescribing is a vital solution. The World Health Organization (WHO) *Medication Without Harm – Global Patient Safety Challenge on Medication Safety*, launched in 2017, aims to reduce severe, avoidable medication-related harm by 50% in 5 years. The WHO describes the importance of considering deprescribing in medication reviews and attending to deprescribing as robustly as prescribing [37]. Likewise, the Australian and Scottish governments and the Lown Institute in America have identified the importance of reducing polypharmacy and educating and training health professionals to reduce medication overload [38–40]. Since 2015, the Canadian Deprescribing Network has been working both nationally and provincially with the public, healthcare professionals, and policymakers to raise awareness about medication safety, deprescribing, and promoting increased access to safer pharmacologic and non-pharmacologic therapies [41, 42]. Other national and international deprescribing networks have also been launched to address the growing concerns regarding aging, polypharmacy, and medication-related harm [43–45].

It is well documented that a number of barriers to deprescribing exist [46–51]. Physicians, pharmacists, nurses, and other healthcare professionals have identified knowledge and skill deficits regarding polypharmacy management, PIM use, balancing medication benefits and harms, and difficulties recognizing ADEs and prescribing cascades. These and other deprescribing concepts are beginning to be taught in some undergraduate programs, but implementation is inconsistent and non-standardized; learners continue to express low confidence and self-efficacy for deprescribing [47, 52–59].

To prepare interdisciplinary teams to deprescribe, we must address these deficits by consistently teaching principles, knowledge, and skills required for effective evidence-based deprescribing. While physicians, pharmacists, and nurses prescribe, deprescribe, dispense, administer, and monitor medications, other healthcare professionals can also identify patients who may benefit from medication review, deprescribing, and non-pharmacological approaches to care [60–67]. Ideally, a structured, robust curriculum within each respective healthcare program should present prescribing principles and assessments to ensure the implementation of appropriate deprescribing practices.

Currently, deprescribing competencies are taught and assessed to varying degrees (or not at all) within healthcare curricula across Canada. A 2018 international symposium on deprescribing education [68] acknowledged that deprescribing skills are not consistently taught or assessed in many countries. Subsequently, members of the Canadian Deprescribing Network’s Healthcare Providers Education Committee, an interprofessional group, sought input from Canadian healthcare professional educators, deprescribing leaders, and clinicians involved in the care of older people. Through an iterative process involving literature review, consultation and several rounds of discussion and revision and group consensus, we developed this position paper to provide guidance on teaching and assessing deprescribing as part of a continuum of appropriate prescribing within an interprofessional context.

This paper targets educators in medicine, pharmacy, and nursing involved in the design and delivery of entry-to-practice (pre-licensure) programs and organizations that accredit these. It also supports the education of other healthcare professionals (e.g., physiotherapists, occupational therapists, dietitians, dentists, speech-language pathologists, social workers) who can identify medication safety concerns or adverse drug effects, and who may recommend non-pharmacological approaches that reduce reliance on medications [61].

The objective of this paper is to provide a deprescribing competency curriculum framework for medicine, nursing, and pharmacy entry-to-practice degree programs. To enable educators to systematically integrate and assess deprescribing knowledge into their teaching and clinical practices, we propose a draft competency framework, options for learning outcomes at various levels, teaching and assessment strategies, and a toolkit of practical resources for curricular and experiential learning. We anticipate that this work will be applicable internationally although it is written from a Canadian perspective. Much of the polypharmacy and deprescribing research and clinical practice initiatives emerge from work in geriatric medicine. Therefore, this paper focuses on deprescribing in the context of care of older people, though we encourage curriculum developers to consider components of the framework in relation to other populations. Ultimately, our goal is to have health professional educators use this framework to identify and implement opportunities for their students to attain competencies in deprescribing.

## Proposed Deprescribing Competency Framework

Deprescribing should be aligned with good prescribing. Several Canadian regulatory bodies, educators, and researchers have adopted the Royal Pharmaceutical Society’s (RPS)* Competency Framework for All Prescribers*, which lists ten competencies for prescribing: six address consultation and patient-centered care and four relate to prescribing governance [69]. The Royal College of Physicians and Surgeons of Canada (RCPSC) developed competencies for prescribing in their *Prescribing Safely Canada* initiative, with ten competencies, seven of which relate to patient and caregiver, and three related to system issues, ethics, and practice reflections [70]. Our draft deprescribing competency framework aligns with the prescribing competencies from both the RPS and RCPSC.

To inform the framework, we began with the deprescribing process outlined by Reeve et al., expanding their five-step process to encompass seven general competencies [71]. These include gathering and interpreting a patient’s medication history and clinical information in the context of patient-specific factors, using tools that help identify PIMs, weighing the potential benefit and harm of continuing or deprescribing medications, using shared decision-making to make decisions about deprescribing, communicating deprescribing and monitoring plans, and monitoring progress and outcomes. The seven statements describe the general competencies for consideration by curriculum planners and are expanded upon in Table 1 [1, 69, 70, 72–77] with descriptions of the required knowledge and skills. We recognize that the competencies may need to be individualized for different programs, but importantly, they begin the conversation about how to integrate deprescribing competencies into healthcare professional education. We also recognize that many health professional programs are tightly scheduled, and it may seem challenging to determine if or how these competencies might be integrated in a busy program. To facilitate this process, we have included those RCPSC and RPS competencies that align with the deprescribing competencies in Table 1, supporting the approach to co-teach these concepts. We have also provided an example of a process that could be used to assess current curricula for consistencies and gaps (Appendix 1).

**Table 1:** Proposed entry-to-practice competencies to guide deprescribing curricula for pre-licensure healthcare professional learners in Canada [1, 69, 70, 72–77]. All healthcare professional curricula are guided by patient care competencies specific to their profession. The proposed competencies below are built upon, or highlighted from, the foundational professional competencies, specific to deprescribing, and are intended to be applied in collaboration with patients and/or their family/care partners. Both the RCPSC [70] and the RPS [69] prescribing frameworks were reviewed in the development of these competencies (RCPSC, Royal College of Physicians and Surgeons of Canada Prescribing Safely Canada Initiative; RPS, Royal Pharmaceutical Society’s Competency Framework for All Prescribers)

**Competency 1: Conduct a comprehensive patient medication history** Aligns with: **RCPSC Competency 1**: Perform a comprehensive assessment of the patient to identify a therapeutic target **RPS Competency 1**: Assess the patient	
**Knowledge required**	**Skills required**
The healthcare professional learner must know the importance of gathering the following: 1. Name of each medication and substance (prescription and non-prescription, including vitamins, supplements, natural health products, herbal products and other substances – e.g., use of alcohol, caffeine, cannabis) currently used or used in the past 2. Name of prescriber, medication indication, dose and frequency, prescribed directions, how actually used, duration of use of each of the above 3. Allergies, side effects, intolerances and contraindications to medications and substances 4. Relevant medical history including pharmacological and non-pharmacological approaches to management of medical conditions 5. Patient’s/care partner’s reason for medication use and expectations, impression of effectiveness or side effects and reasons for stopping or dose reduction for each of above 6. Patient’s/care partner’s beliefs, values, goals of care and perspectives regarding medication use and medical conditions 7. Laboratory, diagnostic imaging, or other test results that support effectiveness or potential for side effects of each of the above 8. Compliance/adherence assessment, including what happens when medications (as above) are missed 9. Medication-related expenses 10. Regimen feasibility and functional considerations for patient (e.g. swallowing) The healthcare professional must know and understand the appropriate use of tools and strategies to conduct Best Possible Medication Histories, Medication Reconciliation, Clinical Medication Review and Medication Management	The healthcare professional learner demonstrates that they are able to: 1. Establish and maintain a therapeutic relationship with the patient and family/care partners, demonstrate understanding of patient’s values, beliefs and goals of care related to medication use and their medical conditions 2. Establish and maintain a relationship with the patient’s interprofessional care team 3. Gather and document reliable medication and health condition information, which may require obtaining information from the patient, family/care partners, medical record, family physician, specialist consults, nursing notes, and/or pharmacist/pharmacy records 4. Use effective communication in accord with patient need, such as consideration being given for language difficulties, sensory impairment, speech issues, cognitive ability and diverse backgrounds to ensure accuracy of information

**Competency 2: Interpret relevant information in the context of desired therapeutic outcomes and goals of care** Aligns with: **RCPSC Competency 2:** Consider optimal pharmacologic and non-pharmacologic options; some components of: **Competency 3:** Prescribe medications appropriate to the patient’s diagnosis, considering cost and risk of benefit and harm, and **Competency 5:** Reach shared decision on medication use and monitoring with the patient and/or family **RPS Competency 2:** Identify evidence-based treatment options	
**Knowledge required**	**Skills required**
The healthcare professional learner must know: 1. Optimal pharmacological and nonpharmacological options to managing the patient’s medical conditions 2. Pharmacokinetic and pharmacodynamic changes that occur with age, multimorbidity, frailty 3. Atypical presentations of medical conditions or medication safety events that may occur with aging 4. Evidence related to specific medication use 5. Limitations of evidence for effectiveness with older age, frailty, multimorbidity and end-of-life 6. Evidence related to impact of multiple medication use (e.g., anticholinergic drug burden, polypharmacy) on patient’s health and medical conditions 7. How prescribing cascades develop and how they can be investigated and managed 8. Appropriate therapeutic success indicators/goals/targets with older age, frailty, multimorbidity and at end-of-life 9. Barriers to effective medication taking or risks for non-adherence to medications	The healthcare professional learner demonstrates that they are able to: 1. Interpret the literature/evidence to support therapeutic efficacy and safety of the options available 2. Conduct patient assessment with focused attention to indications for drug therapy (for treatment and/or prevention) 3. Interpret medication indication, effectiveness, safety and patient adherence based on patient specific factors (e.g., age, multiple medication use, frailty) 3.1. Recognize potentially inappropriate medications for the patient’s conditions 3.2 Determine/interpret effectiveness of the medication 3.3 Identify appropriate dose and duration of therapy 3.4 Identify potential for adverse effects 3.5 Identify whether a medication is contributing to a current condition (sign, symptom) that may or may not be being treated (e.g., may be responsible for a prescribing cascade) 3.6 Identify potential for drug-drug or drug-disease interactions 4. Identify relevant and evidence-based therapeutic targets and goals of care based on patient specific factors (e.g., age, multiple medications, frailty, neurocognitive disorder, life expectancy, patient goals, other comorbid conditions)

**Competency 3: Identify medications that are no longer necessary, may have more harm than benefit, or are otherwise potentially inappropriate**	
**Knowledge required**	**Skills required**
The healthcare professional learner must know: 1. How potentially inappropriate medications (PIMs) are defined 2. The tools available to identify PIMs in older adults (e.g., AGS Beers Criteria, Screening Tool of Older Person’s Prescriptions (STOPP) criteria, anticholinergic burden scales Medication Appropriateness Index) 3. The tools available to identify potentially inappropriate medications in those with frailty/dementia or in palliative care (e.g., STOPP-Frail, Good Palliative-Geriatric Practice algorithm) 4. How to evaluate medications without an indication (condition resolved or unconfirmed), or with questionable efficacy, altered risk, related to a prescribing cascade, causing an adverse drug event, or potential for future harm	The healthcare professional learner demonstrates that they are able to: 1. Identify medications that have greater risk of harm versus benefits in older adults 2. Assess a patient’s medication regimen for PIMs by applying various tools that can be used to determine if a patient’s medications are potentially inappropriate 3. Describe potential adverse effects of the patient’s medications and apply the concept of atypical presentation in older adults, or adverse events that can occur in a multimorbid patient with a complex regimen 4. Use clinical judgement integrated with knowledge of disease management, geriatric principles of care, pharmacokinetics and pharmacodynamics, to determine if a medication not on an explicit tool/list could be inappropriate for a particular patient (e.g., potentially unnecessary or possibly causing more harm than benefit) 5. Determine if any of the patient’s medications, dosage, or administration are potentially inappropriate and provide the rationale

**Competency 4: Assess deprescribing potential of each medication by weighing benefits and harms** Aligns with: **RCPSC Competency 2**: Consider optimal pharmacological and nonpharmacological options, **Competency 3**: Prescribe medications appropriate to the patient’s diagnoses, considering cost and risk of benefit and harm **RPS Competency 2**: Identify evidence-based treatment options available for clinical decision-making, **Competency 3**: Present options and reach a shared decision	
**Knowledge required**	**Skills required**
The healthcare professional learner must know: 1. The evidence for the harms and benefits of continuing each medication 2. The evidence for the harms and benefits of deprescribing each medication 3. The possibility of an adverse drug event as an etiology for any new sign or symptom 4. The non-pharmacologic options for prevention and management of disease 5. The combined harms of all medications that the patient is taking (e.g., added falls risk, anticholinergic burden) 6. The impact of patient complexity (e.g., patient’s co-morbidities, dysphagia, appropriate medication formulation, if patient needs palliative care) and the logistics of how the care team functions (e.g., multiple prescribers, consultation by numerous specialists, involvement of home care), that may affect the optimal management of the patient’s medication therapy 7. Populations at increased risk of adverse drug events (e.g., those with renal failure, dementia, frailty, multiple-comorbidities, patients in long-term care facilities) 8. Factors that may limit the benefit of medications (e.g., those with short life expectancy, prevention therapies that require long duration for effect) 9. The time needed for medication to achieve effect, known as time to benefit	The healthcare professional learner demonstrates that they are able to: 1. Interpret harms and benefits of continuing or deprescribing medications for a particular patient 2. Identify when non-pharmacologic options for prevention and management of disease would be appropriate for a patient and if they could facilitate deprescribing 3. Review the value of preventive medications, or those for symptomatic treatment versus disease-modifying treatment for the patient 4.Assess the implications of polypharmacy (e.g., medication regimen complexity, pill burden, drug interactions) on the patient 5.Assess the patient’s frailty, cognition and multimorbidity (i.e., interacting medical conditions) 6.Assess the benefits and harms of the medication, taking into account the patient’s health goals, values, preferences and beliefs, and generalizability of evidence for use of the medication 7.Assess time to benefit estimated from data from best available evidence and compare with the patient’s life expectancy

**Competency 5: Decide whether deprescribing a medication is appropriate using shared decision-making** Aligns with: **RCPSC Competency 4:** Provide medication-relevant information ensuring patient/family understanding and ability to access. **Competency 5**: Reach a shared decision on medication use and monitoring with the patient and/or family **RPS Competency 3**: Present options and reach a shared decision, **Competency 4**: Prescribe, **Competency 5**: Provide information, **Competency 7**: Prescribe safely	
**Knowledge required**	**Skills required**
The healthcare professional learner must know: 1. The four steps of the shared decision-making process for deprescribing (i.e., create awareness for options, discuss potential benefits/harms of options, explore patient preferences, work with patient to make a decision) 2. The role of capacity in making informed medication decisions	The healthcare professional learner demonstrates that they are able to: 1. Apply the concept of shared decision-making to deprescribing discussions with patients and/or family/care partners (including talk about benefits, harms of pharmacologic and non-pharmacologic options) 2. Use appropriate communication skills to engage with the patient and/or family/care partners within a shared decision-making framework to determine if a medication should be continued, withdrawn or the dose reduced 3. Discuss with patients and/or family/care partners regarding their values, preferences, beliefs and goals of care surrounding continued medication use versus deprescribing 4. Elicit which medications are important for the patient to continue and explore why 5. Take into account the patient’s capacity to make medical decisions when eliciting describing preferences 6. Demonstrate the ability to balance family/care partner input and concerns with patient care decisions 7. Make a determination, along with the patient’s and healthcare team’s input as to whether each medication should be continued or deprescribed, or whether dosing regimen could be simplified

**Competency 6: Design, document, and share a deprescribing and monitoring plan** Aligns with: **RCPSC Competency 6:** Monitor and review the patient’s medications and adherence at each encounter, aiming to optimize the regimen, **Competency 7**: Prescribe carefully with attention to medication safety, **Competency 8**: Prescribe responsibly and ethically within legal and regulatory frameworks **RPS Competency 5**: Provide information, **Competency 6**: Monitor and review, **Competency 7**: Prescribe safely, **Competency 8**: Prescribe professionally; **Competency 10:** Prescribe as part of a team	
**Knowledge required**	**Skills required**
The healthcare professional learner must know: 1. Medications commonly associated with adverse drug withdrawal events and the timing of withdrawal based on pharmacodynamics, pharmacokinetics and other medication factors 2. The order, priority and timing for deprescribing when more than one medication is to be withdrawn. (e.g., based on potential for harm, patient preference, pharmacodynamics) 3. Enablers or challenges of medication use that may impact on medication reduction (e.g., patient's use of dosage administration aids, caregivers involved in medication administration) 4. Non-pharmacologic options to reduce reliance on medication 5. How to manage adverse drug withdrawal events 6. Resources and sources for evidence-based deprescribing guidelines, algorithms, and other tools 7. Communication strategies to increase patient/care partner comfort with the process of deprescribing 8. The activities and steps required for implementation of deprescribing, and how the interprofessional team may collaborate to ensure these are accomplished 9. The key components of written and electronic documentation (including outlining risks and benefits and monitoring plans)	The healthcare professional learner demonstrates that they are able to: 1. Decide which medications should be tapered and which can be discontinued without tapering 2. Apply evidence-based deprescribing guidelines, algorithms, and other tools when appropriate 3. Determine whether a non-pharmacological intervention would facilitate deprescribing 4. Determine the best dosing/tapering and timing approach to deprescribing 5. Develop a process to prioritize multiple medications for deprescribing 6. Develop a monitoring plan for deprescribing (i.e., symptoms and signs that should be monitored during deprescribing, how often and for how long, and by whom) 7. Take responsibility with the health care team on the process of deprescribing, clarifying who should monitor the patient and when and for how long 8. Identify those external to the immediate team who support the patient and need to be notified/engaged regarding the deprescribing process 9. Outline the deprescribing and monitoring plan collaborating with the patient, including the family/care partners as appropriate 10. In collaboration with the team, identify and educate regarding non-pharmacological options to support deprescribing including minimizing the return of symptoms or adverse drug withdrawal events 11. Document a patient care plan for deprescribing (including rationale and process) in a chart or electronic health record and distribute interprofessional communication as appropriate 12. Provide documentation to patient and family/care partners in lay language

**Competency 7: Monitor patient progress and provide support** Aligns with: **RCPSC Competency 6**: Monitor and review the patient’s medications and adherence at each encounter, aiming to optimize the regimen, **RPS Competency 6:** Monitor and review, **Competency 9**: Improve prescribing practice, **Competency 10**: Prescribe as part of a team	
**Knowledge required**	**Skills required**
The healthcare professional learner must know: 1. The timing and severity of the clinical presentation(s) of adverse drug withdrawal events, as well as management strategies 2. The signs and symptoms of the patient’s medical conditions or possible indications for medications 3. The signs and symptoms for monitoring for improvement of adverse effects caused by medications and of the ‘reversal’ of drug interactions	The healthcare professional learner demonstrates that they are able to: 1. Monitor for adverse drug withdrawal events, return of condition, reversal of drug interactions 2. Monitor for benefits of deprescribing (e.g., resolution of adverse drug events, improved compliance) 3. Manage adverse drug withdrawal events 4. Determine when to slow or stop the deprescribing process and/or determine the need for other therapies 5. Identify when the patient should restart therapy and how this will be done 6. Identify the supports the patient will require throughout the deprescribing process 7. Identify the timeline for having a patient visit/follow-up 8. Document on progress and outcomes (e.g., medication discontinued, dose reduced or withdrawal attempted with reasons for failure)

To make decisions about deprescribing, healthcare professionals must understand a patient’s medication experience. Medication reconciliation can identify medications without a clear/current indication. Explicit tools such as STOPP/START [73] and AGS Beers Criteria® [1] and implicit tools such as the Medication Appropriateness Index [75] can be used to identify potentially inappropriate or unnecessary medications. Reviewing the patient’s clinical conditions, functional status, and laboratory tests can guide decisions regarding medication choice, benefit/harms, dosing, interactions, and contraindications.

A shared decision-making process supports patient and healthcare professional discussions about the balance of potential benefit and harm for each medication [78]. This can elucidate medications that may no longer be appropriate and help prioritize these for deprescribing [79]. Healthcare professionals must be able to elicit patient values and beliefs about their health and their goals of care, and incorporate this information with their clinical knowledge of the medical conditions and medications and the context in which care is being delivered to inform priorities and plan for how deprescribing will take place [80].

Implementing deprescribing requires both technical knowledge and cognitive skills. These include understanding prescription changes, regulatory requirements, and communication/documentation skills. Cognitive skills include the ability to prioritize, think critically, use emotional intelligence for interactions with patients, and work within an interprofessional team.

The following competencies, to be applied in collaboration with the patient and/or their family/care partners, are proposed for deprescribing:

1. Conduct a comprehensive patient medication history
2. Interpret relevant information in the context of desired therapeutics outcomes and goals of care
3. Identify medications that are no longer necessary, may have more harm than benefit, or are otherwise potentially inappropriate
4. Assess the deprescribing potential of each medication by weighing benefits and harms
5. Decide whether deprescribing a medication is appropriate using shared decision-making
6. Design, document, and share a deprescribing and monitoring plan
7. Monitor patient progress and provide support.

## Consider Interprofessional Education

Based on their scope of practice, each entry-to-practice curriculum emphasizes different knowledge and skills related to medication use. All learners must have the requisite competencies related to health and evidence-informed practice and demonstrate the ability to develop a therapeutic relationship with the patient, engage them in their care, and work interprofessionally within their scope of practice.

Healthcare professionals in medicine, pharmacy, and nursing identify that they have a clear responsibility in medication management [61]. This includes identifying opportunities for deprescribing, engaging in shared decision-making, implementing the deprescribing plan, and following up on the deprescribing process.

Effective deprescribing leverages each healthcare professional’s strengths and unique perspective on patient care. Teaching deprescribing provides an opportunity for interprofessional education demonstrating how, for example, physicians, pharmacists, nurses, dentists, dieticians, physiotherapists, social workers, and others, can collaborate with patients and family/care partners. It is important to understand how an interprofessional team can function within various healthcare settings to leverage each professional’s expertise and scope of practice to improve medication use and safety.

## Education Strategies

Deprescribing can be included early, midway, and later within programs. To understand the prescribing-deprescribing continuum, key concepts should be introduced early and built upon throughout the curriculum in a sequential manner. The competencies (Table 1) in this document focus on the knowledge and skills that learners completing their entry-to-practice degrees should be able to perform successfully. The learning outcomes (Table 2) are more specific and measurable and can be integrated into specific lesson plans or course syllabi. The expected level of competency for deprescribing activities may vary and should be determined by the health professional program, for example, whether a specific competency should be taught at an advanced or intermediate level, depending on the role of that health professional in medication management.

**Table 2:** Teaching and assessment of knowledge and skills related to deprescribing in pre-licensure healthcare professional curriculum

**Proposed learning outcomes**		
**Introductory/early learner**	**Mid-level learner**	**Advanced learner**
Identify individuals and groups such as older adults who are vulnerable to medication-related problems Describe the prevalence of medication-related harm in older adults Provide definitions for ‘polypharmacy’ and ‘deprescribing’ Describe the criteria used to define potentially inappropriate medications (PIMs) List tools used to identify PIMs Identify high risk medications in a given patient case Demonstrate an understanding of challenges faced by older adults taking multiple medications	Discuss the process used to guide prescribing in multimorbid/complex patients Identify patient related factors that increase the risk for medication-related problems, including polypharmacy Apply tools to identify PIMs Describe patient beliefs that may impact on a patient’s medication related decisions Describe how the patient’s family/care partners may play a role in polypharmacy and deprescribing management Identify and apply tools to make decisions about and implement deprescribing Determine benefits and harms of deprescribing a medication Describe the role of different healthcare professionals in managing polypharmacy and implementing deprescribing Demonstrate appropriate documentation and communication strategies to execute a deprescribing plan Examine the ethics and conflicts of interest for healthcare professionals in deprescribing Evaluate advocacy efforts regarding medication safety, prescribing, and deprescribing efforts in older adults	Critique tools used to identify PIMs and to deprescribe medications Use a systematic process for deprescribing Identify patients who should be prioritized/targeted for deprescribing Consider the patient’s preferences, care goals and life expectancy in deciding whether to continue or deprescribe a medication Using shared decision-making, negotiate a deprescribing plan for the medication with the patient and his/her caregivers Design care plans and make deprescribing decisions for high-risk patient groups (including those with dementia, with frailty, receiving palliative care, with multimorbid conditions) Identify opportunities for deprescribing in all settings where care is provided (including acute care, primary care, long-term care) Utilize cognitive dissonance or other communications strategies to promote deprescribing Appraise public health policy relating to medications and deprescribing in older adults

**Examples of teaching and learning activities**		
**Introductory/early learner**	**Mid-level learner**	**Advanced learner**
Practice taking medication histories Interview patients with lived experience to discuss medication experiences related to polypharmacy and deprescribing Document medication related problems Discuss written simulated case studies Take-home simulation activity such as mimicking being on a complex medication regimen to understand adherence Write a journal entry regarding a simulated medication experience	Patient and family members sharing their lived experiences and challenges regarding medication burden and medication related harm through presentations or panel discussions Think-pair-share to identify priorities for deprescribing Group presentations of topics on deprescribing Active discussions and debate on deprescribing related topics Gamification to apply deprescribing concepts to patient cases Interprofessional discussion regarding each health care profession’s role and responsibilities in implementing and following up on deprescribing plans in complex patients Discussion on practicalities of implementation of deprescribing in a busy practice where teams are not co-located	Using interactive cases, ask learner to identify critical pieces of information related to polypharmacy and deprescribing Learner asked to critique relevant articles through journal clubs Learner/group debates on polypharmacy and deprescribing related topics Learner to work through simulated interprofessional case studies Learner to collaborate with primary care /family health team regarding focused deprescribing projects Group projects for business plans such as development of a new clinical service related to medications and deprescribing Reflective journaling activities regarding challenging cases related to medication problems and deprescribing Group projects in the policy area, guided/precepted by public health policy makers within the Ministry of Health or within Schools of Public Health

**Examples of assessment strategies**		
**Introductory/early learner**	**Mid-level learner**	**Advanced learner**
Multiple choice questions, short answer quizzes or examinations Assess paper case study care plans Assess critique of a journal article on polypharmacy or deprescribing Assess reflective essay regarding the burden of polypharmacy	Assess presentations (individual, group) of paper or simulated case studies or journal article reviews related to the evidence for deprescribing Assess care plans developed for simulated patient cases involving minimal/moderate complexity Assess longitudinal case studies (simulated or actual patients) requiring learner to develop and implement care plans for deprescribing Assess ability to discuss and debate policies or practices that relate to medication problems and deprescribing Assess reflective essay regarding interpretation of evidence, identification of gaps in the literature, and application to decision-making for deprescribing	Assess care plans for simulated or actual patient cases involving frail older adults with multiple comorbidities Assess performance of direct patient care related activities (during advanced experiential practice, rotations, clerkships, practicums) Assess Individual and Team based Observed Structured Clinical Exam (OSCE) Assess evidence-based review of deprescribing intervention Assess the development and implementation of team-based design and testing of deprescribing processes in primary care or other settings Assess a dossier or portfolio of case studies demonstrating integration of deprescribing activities Assess a position paper on a policy or practice related to implementation of deprescribing

*PIMs* potentially inappropriate medications, *OSCE* Observed Structured Clinical Exam

Deprescribing content can be taught as a standalone concept/module/course or progressively integrated into various courses throughout the program and included within experiential education. Standalone content may be appropriate for curricula where medication management is not a focus but where learners would benefit from understanding how some patient symptoms may be related to medications and may resolve with deprescribing. In such instances, highlighting their contribution toward reducing medication harm and supporting non-drug interventions is important.

Implementing effective deprescribing requires the application of knowledge regarding biological, pharmaceutical, and social sciences and a broad understanding of medication use behaviors, medication processes, pharmacokinetics, pharmacodynamics, team dynamics, shared decision-making, regulations, and policies. As polypharmacy is highly prevalent in older adults, much of our knowledge about deprescribing is informed by this population. As such, it is logical to include geriatrics courses that teach deprescribing; however, as we have outlined, principles should be introduced early on and carried throughout the curriculum.

Table 2 provides details, where we have applied models for learning frameworks and curriculum design [81, 82] to propose learning outcomes at different levels, examples of teaching and learning activities, and examples of assessment strategies. Ensuring learners have numerous, scaffolded, thoughtful learning and assessment activities throughout their programs, with meaningful interprofessional touchpoints, will support the development of competency in deprescribing.

### Introductory/Early Learner

All healthcare professional learners begin to develop professional identity through understanding their scope of practice. At this stage, they require an understanding of the epidemiology of common health conditions and how such conditions may be caused or exacerbated by medications.

Medicine, pharmacy, and nursing programs should introduce learners to patient care and medication use processes, including how decisions related to prescribing and deprescribing are made. Such concepts should be taught in the context of effective communication within a shared decision-making framework. Learners must be able to gather information related to medication use and identify medications that may be inappropriate.

### Mid-level Learner

All healthcare professional learners should be introduced to principles of geriatric medicine and made aware of common geriatric syndromes and medication-related problems, including drug interactions and adherence challenges. It is essential to advocate for a patient-centered approach, including the importance of including family/care partners in decision-making.

Approaches to interprofessional practice should be included in all curricula. Using the Canadian Interprofessional Health Collaborative (CIHC) Interprofessional Framework [83] and the domains of interprofessional communication, patient- and family-centered care should be introduced, emphasizing how this can support discussions regarding medication use.

Learners in medicine, pharmacy, and nursing should have specific curriculum requirements relating to geriatrics including common medication-related problems and physiological changes that affect medications with aging. They should be able to elicit a patient’s goals of care and determine if they align with their medications. Education should build on introductory learning to include the application of tools to identify polypharmacy, PIMs, and resources/tools to guide medication decision-making. Learners should be able to describe the deprescribing process, evidence related to both benefits and harms of medications, and evidence supporting deprescribing when available. Discussions of ethical challenges relating to medication problems in older adults, including the ethics of gaps in evidence, should be introduced. Learners should be able to apply deprescribing concepts to a simulated case of a patient with minimal disease burden/complexity.

### Advanced Learner

All learners should be able to describe why and how older adults and other groups may be more vulnerable to medication problems and develop an understanding of how to provide care for those with late-stage chronic disease, terminal disease, dementia, and frailty, and those at the end-of-life. Additionally, learners should understand how care is provided in various settings (e.g., long-term care, acute care, outpatient, primary care) and the implications of deprescribing in these contexts, including unique system barriers, processes, and patient needs within different settings.

Medicine, pharmacy and nursing learners should be able to critically assess the levels of evidence, validity, strengths, and weaknesses of various tools used to identify PIMs and deprescribe medications, and determine the appropriate situations for the tools’ application. Learners should be able to prioritize medications for deprescribing and develop and implement a care plan that includes monitoring and support. Learners should be able to approach deprescribing given an integrated case study involving an older adult with complex multimorbid conditions. Complexity factors may also include ethical concerns, family dynamics, cultural differences, special or vulnerable populations. The curriculum should guide learners on how best to advocate for appropriate deprescribing, especially in older adults, and to understand safe medication practices.

## Teaching and Assessment Strategies

Each healthcare profession has a unique culture and context for curriculum delivery and assessment, and each program may have different access to resources.

General guidelines for teaching deprescribing include:

- Introduce deprescribing concepts early and emphasize that deprescribing is a continuum of prescribing
- Provide education throughout all years of the program using progressively complex cases to enable learners to develop knowledge and skills related to medications and deprescribing
- Increase the complexity of deprescribing scenarios by introducing different healthcare settings, family/care partners, team members, ethical considerations, and clinical characteristics
- Structure deprescribing discussions in relation to all therapeutic areas and within curriculum relating to the patient’s social and health system context
- Plan and integrate deprescribing concepts across interprofessional education programs
- Incorporate teaching and learning strategies such as didactic, active learning, simulation, and experiential education

General guidelines for the assessment of deprescribing include:

- Provide learners with both formative and summative feedback regarding deprescribing practice cases and assignments
- Assess foundational concepts through multiple choice questions or short answers
- Consider a range of approaches and decisions regarding deprescribing that are supported with rationale and development of care plans
- Employ Objective Structured Clinical Examinations (OSCE) and Team OSCEs (TOSCE) to assess the application of basic and complex knowledge at different points in the program, from early to advanced levels
- Integrate deprescribing concepts that can be assessed in other related courses that include communication activities, application of ethical principles, or assessment of evidence
- Include specific objectives related to prescribing and deprescribing in experiential education/practicum and ensure assessment is conducted by preceptors (e.g., appropriately weighing of potential benefit and harm, communication, documentation, follow-up)
- Ensure feedback from multiple sources, including patients and family/care partners, simulation facilitators, professors, near peers (same year, same program), senior peers (higher year, same program), and interprofessional team peers

## A Call to Action

Deprescribing is best accomplished within an interprofessional practice that is patient-centered and informed by shared decision-making. Based on the seven deprescribing competencies outlined, educators should identify learning objectives to be attained through both uni- and inter-professional education. The specific knowledge and skills taught within programs will be dependent on the perceived gap between the suggested competencies and performance of graduates from the program.

Curricular leaders, national health professional faculty associations, and accrediting bodies must take the following steps, within suggested timeframes, to ensure that learners in medicine, pharmacy, and nursing attain the required competencies in deprescribing and that all healthcare professionals are able to recommend interventions, including non-pharmacological management, that reduce reliance on medications:

In the next year:

- Examine what needs to happen within your profession, your program, and across interprofessional curricula to ensure graduates are trained to undertake or support deprescribing in their practice

In the next 2 years:

- Map the curriculum to determine where, when, and how deprescribing competencies are included in current programming, including how they are taught and assessed and identify areas for inclusion
- Develop a plan to address curricular gaps
- Create opportunities within curricula to implement deprescribing competencies and determine how these will be taught and assessed
- Utilize practical tools (Table 3) throughout the curriculum

In the next 4–6 years:

- Evaluate the core deprescribing competencies of graduates to determine the effectiveness of curricular changes

Annually:

- Share learnings and outputs (e.g. curricular innovations, continuous professional development opportunities, prescribing and deprescribing competency frameworks) with the Canadian Deprescribing Network (info@deprescribingnetworks.ca) to facilitate sharing, networking and collaboration across Canada.

**Table 3:** Deprescribing Resources Toolkit

General information and resources about deprescribing	
• Canadian Deprescribing Network website (resources for health professionals and the public) www.deprescribingnetwork.ca	
• Bruyère Deprescribing Research Team website www.deprescribing.org/ and YouTube channel (whiteboard videos about deprescribing guidelines, testimonials, and webinars) www.youtube.com/channel/UCwqOu26_nAMmUyb3fyKxBbw	
• Scottish Government Polypharmacy Model of Care Group. Polypharmacy Guidance, Realistic Prescribing 3rd Edition, 2018. Scottish Government website www.managemeds.scot.nhs.uk/ and guide www.therapeutics.scot.nhs.uk/wp-ontent/uploads/2018/04/Polypharmacy-Guidance-2018.pdf	
• Deprescribing: A Practical Guide: NHS Derby and North Derbyshire Clinical Commissioning Group Medicines Management Team. www.derbyshiremedicinesmanagement.nhs.uk/assets/Clinical_Guidelines/clinical_guidelines_front_page/Deprescribing.pdf	

Educational resources for teaching of deprescribing competencies	
• Polypharmacy and Deprescribing online module (Bruyère Continuing Care) www.bruyere.org/en/polypharmacy-deprescribing	
• Case reports with worksheets and instructions for interprofessional case discussions about polypharmacy and deprescribing:	
- Farrell B, Eisener-Parsche P, Dalton D. Turning over the rocks – The role of anticholinergics and benzodiazepines in cognitive decline and falls. Can Fam Physician. 2014;60:345–350. www.cfp.ca/content/60/4/345 - Farrell B, Monahan A, Thompson W. Revisiting ongoing medication use in a frail 93 year old experiencing possible adverse effects. CMAJ. 2014;186:445–449. www.cmaj.ca/content/186/6/445 - Farrell B, Shamji S, Dalton D. Managing chronic disease in the frail elderly – More than just adhering to clinical guidelines. Can Pharm J. 2014;147:89–96. www.ncbi.nlm.nih.gov/pmc/articles/PMC3962056/ - Farrell B, Monahan A, Ingar N. Identifying and managing drug-related causes of common geriatric symptoms. Can Fam Phys. 2014;60:147–153. www.cfp.ca/content/60/2/147 - Farrell B, French Merkley V, Ingar N. Reducing pill burden and helping with medication awareness to improve adherence. Can Pharm J. 2013;146:262–269. https://www.journals.sagepub.com/doi/10.1177/1715163513500208 - Farrell B, French Merkley, Thompson W. Managing polypharmacy in a 77-year-old woman with multiple prescribers. CMAJ. 2013;185:1240–1245. www.cmaj.ca/content/185/14/1240 - Farrell B, Shamji S, Ingar N. Reducing fall risk while managing hypotension, pain and poor sleep in an 83 year old woman. Can Fam Physician. 2013;59:1300–1305. www.cfp.ca/content/59/12/1300 - Farrell B, Shamji S, Ingar N. Reducing fall risk while managing pain and insomnia: addressing polypharmacy in an 81 year old woman. Can Pharm J. 2013;146:335–341. https://www.journals.sagepub.com/doi/10.1177/1715163513504529	

Tools to help identify potentially inappropriate medications	
*Implicit tools*	• Medication Appropriateness Index: • Hanlon JT, Schmader KE. The medication appropriateness index at 20: Where is started, where it has been and where it may be going. Drugs Aging. 2013;30:893–900. https://pubmed.ncbi.nlm.nih.gov/24062215/ • Examples of Calculators: www.globalrph.com/medcalcs/medication-appropriateness-index-calculator/ • www.cgakit.com/m-2-mai
*Explicit Tools*	Beers Criteria: • American Geriatrics Society 2019 Updated AGS Beers Criteria® for Potentially Inappropriate Medication Use in Older Adults https://onlinelibrary.wiley.com/doi/10.1111/jgs.15767 STOPP/START: • O'Mahony D. STOPP/START criteria for potentially inappropriate medications/potential prescribing omissions in older people: Origin and progress. Expert Rev Clin Pharmacol. 2020;13:15–22. www.pubmed.ncbi.nlm.nih.gov/31790317 • Lavan A, Gallagher P, Parsons C, O'Mahony D. STOPPFrail (Screening tool of older persons prescriptions in frail adults with limited life expectancy): Consensus validation. Age Ageing. 2017;46:600–607. www.academic.oup.com/ageing/article/46/4/600/2948308
*Anticholinergic burden*	• Calculating anticholinergic burden www.acbcalc.com/ • Salahudeen MS, Duffull SB, Nishtala PS. Anticholinergic burden quantified by anticholinergic risk scales and adverse outcomes in older people: A systematic review. BMC Geriatr. 2015;15. www.bmcgeriatr.biomedcentral.com/articles/10.1186/s12877-015–0029-9 • Lisibach A, Benelli V, Ceppi MG, Waldner-Knogler K, Csajka C, Lutters M. Quality of anticholinergic burden scales and their impact on clinical outcomes: A systematic review. European Journal of Clinical Pharmacology. 2021;77:147–162. www.pubmed.ncbi.nlm.nih.gov/33011824/

Examples of guidelines useful for deprescribing	
• Frailty-specific guidelines www.pathclinic.ca/education/clinical-practice-guidelines/	
• Statins: Mallery LH, Moorhouse P, McLean Veysey P et al. Severely frail elderly patients do not need lipid-lowering drugs. Cleve Clin J Med. 2017 Feb;84:131–142. pubmed.ncbi.nlm.nih.gov/28198686/	
• Antidepressants: Mallery L, MacLeod, T, Allen, M, et al. Systematic Review and Meta-analysis of Second-Generation Antidepressants for the Treatment of Older Adults with Depression: Questionable Benefit and Implications for Frailty. BMC Geriatr. 2019;19. bmcgeriatr.biomedcentral.com/articles/10/1186/s12877-019–1327-4	
• Diabetes: Mallery LH, Ransom T, Steeves B, et al. Evidence-informed guidelines for treating frail older adults with type 2 diabetes: From the Diabetes Care Program of Nova Scotia (DCPNS) and Palliative and Therapeutic Harmonization (PATH) Program. J Am Med Dir Assoc. 2013;14:801–808. www.sciencedirect.com/science/article/pii/S152586101300460X?via%3Dihub	
• Hypertension: Mallery L., Allen M., Fleming I., Kelly K., Bowles S., Duncan J., Moorhouse P. Promoting higher blood pressure targets for frail older adults: A consensus guideline from Canada. Cleveland Clin J Med. 2014; 87:427–37. pubmed.ncbi.nlm.nih.gov/24987044/	

Evidence-based deprescribing guidelines	
• Pottie K, Thompson, W, Davies S, Grenier J, Sadowski CA, Welch V, Holbrook A, Boyd C, Swenson R, Ma A, Farrell B. Deprescribing benzodiazepine receptor agonists: Evidence based clinical practice Guideline. Can Fam Physician. 2018; 64:339–351. www.cfp.ca/content/64/5/339	
• Reeve E, Farrell B, Thompson W, Herrmann N, Sketris I, Magin P, Chenoweth L, Gorman M, Quirke L, Bethune G, Forbes F, Hilmer S. Evidence-based Clinical Practice Guideline for Deprescribing Cholinesterase Inhibitors and Memantine. Sydney: University of Sydney. 2018. www.sydney.edu.au/medicine/cdpc/resources/deprescribing-guidelines.php	
• Bjerre LM, Farrell B, Hogel M, Graham L, Lemay G, McCarthy L, Raman-Wilms L, Rojas-Fernandez C, Sinha S, Thompson W, Welch V, Wiens A. Deprescribing antipsychotics for behavioural and psychological symptoms of dementia (BPSD) and insomnia: Evidence-based clinical practice guideline. Can Fam Physician. 2018;64:17–27. www.cfp.ca/content/64/1/17	
• Farrell B, Black C, Thompson W, McCarthy L, Rojas-Fernandez C, Lochnan H, Shamji S, Upshur R, Bouchard M, Welch V. Deprescribing antihyperglycemic agents in older persons: An evidence-based clinical practiced guideline. Can Fam Physician. 2017;63:832–842. www.cfp.ca/content/63/11/832	
• Farrell B, Pottie K, Thompson W, Boghossian T, Pizzola L, Rashid FJ, Rojas-Fernandez C, Walsh K, Welch V, Moayyedi P. Deprescribing proton-pump inhibitors: An evidence based clinical practice guideline. Can Fam Physician. 2017;63:354–364. www.cfp.ca/content/63/5/354	
• Evidence-based deprescribing guidelines app deprescribing.org/news/evaluation-of-a-deprescribing-guideline-mobile-application-2/	

Weighing risk and benefit to help make decisions about prescribing/deprescribing—a few examples	
• PPI Deprescribing Patient Decision Aid www.deprescribing.org/resources/deprescribing-patient-decision-aids • Thompson W, Farrell B, Welch V, Way C, Richardson L, Bjerre L. Continuation or deprescribing of proton pump inhibitors: A consult patient decision aid. Can Pharm J. 2018;152:18–22 •Thompson W, Farrell B, Welch V, Tugwell P, Bjerre L. Should I continue taking my acid reflux medication? Design of a pilot before/after study evaluating a patient decision aid. Can Pharm J. 2017;150:19–23	
• Patient Decision Aids www.decisionaid.ohri.ca/	
• Shared Decision Making in Deprescribing webinar www.youtube.com/watch?v=Ywzhd0cj7Ls	

Publications and tools to help plan deprescribing and monitoring	
• Reeve E. Deprescribing tools: A review of the types of tools available to aid deprescribing in clinical practice. Journal of Pharmacy Practice and Research 2020;50:98–107. onlinelibrary.wiley.com/doi/10.1002/jppr.1626	
• Farrell B, Mangin D. Deprescribing is an essential part of good prescribing. American Family Physician. 2019;99:7–9. www.aafp.org/afp/2019/0101/p7.html	
• Evidence based deprescribing guidelines and algorithmswww.deprescribing.org/resources/deprescribing-guidelines-algorithms/, patient pamphlets and infographics deprescribing.org/resources/deprescribing-information-pamphlets/	
• Graves T, Hanlon JT, Schmader KE, Landsman PB, Samsa GP, Pieper CF, Weinberger M. Adverse Events After Discontinuing Medications in Elderly Outpatients. Arch Intern Med. 1997;157:2205–2210. https://pubmed.ncbi.nlm.nih.gov/9342997/	
• Dalhousie University deprescribing resource: “Toolbox for Healthcare Providers” www.dal.ca/sites/gmr/our-work/fewer-pills-less-risk/toolbox-for-healthcareproviders.html	
• McMaster University/TAPERMD “Resources for Safer Medication Use” www.pimsplus.org and https://tapermd.com/	
• GeriRxFiles (a subscription academic detailing and drug information service based in Saskatchewan, Canada) www.rxfiles.ca/geri	
• MedStopper www.medstopper.com/	
• MedSafer – Working Towards Safer Prescribing www.medsafer.org	
• Primary Health Tasmania Deprescribing Resources www.primaryhealthtas.com.au/resources/deprescribing-resources/	

**Tools to support interprofessional education strategies**	
• Canadian Interprofessional Health Collaborative (CIHC) Framework www.mcgill.ca/ipeoffice/ipe-curriculum/cihc-framework	

## Conclusion

Integrating deprescribing competencies in healthcare curricula requires an intentional and structured approach across all years of the program, focusing on interprofessional collaboration. Learning activities should be active and practical, progressing from early to advanced learner skills and include integration of deprescribing during experiential education. As appropriate, the guidelines for curriculum design and learner assessments in deprescribing, emphasized within a geriatric context, can be integrated throughout all therapeutic content/courses. The process of preparing healthcare professionals to be confident deprescribers relies on a solid foundation within the pre-licensure curriculum. Building this foundation is essential to ensure healthcare professionals are able to deprescribe safely to minimize the personal and societal costs of medication-related harm.

## Supplementary Information

Below is the link to the electronic supplementary material.

Supplementary file1 (DOCX 32 KB)

## Data Availability

Not applicable.
