# Supplementary material for: A Proposed Curricular Framework for an Interprofessional Approach to Deprescribing
Source: Med Sci Educ. 2023 Feb 23;33(2):551–67. doi: 10.1007/s40670-022-01704-9 (PMC10226933; doi:10.1007/s40670-022-01704-9)
Supplement: Supplementary file 1 — Supplementary file1 (DOCX 32 KB) [file 40670_2022_1704_MOESM1_ESM.docx]

**Appendix 1 Incorporating deprescribing competencies into medical school curricula (a Canadian example)**

The following provides suggestions on how a medical school program in Canada could interpret deprescribing competencies and embed them within existing competencies and curricula.

**Introduction and Overview**

Medical school curricula in Canada are developed to align with the CanMEDS Framework [1], whose “overarching goal is to improve patient care”. CanMEDS roles form the basis for the Royal College of Physicians and Surgeons of Canada’s (RCPSC) competencies [2], which include ten prescribing competencies to promote effective prescribing [3].

Deprescribing is a continuum of good prescribing. As such, prescribing competencies already encompass skills that facilitate deprescribing. To ensure further mastery, educators can add deprescribing principles to existing prescribing competencies.

**Knowledge and skills required for deprescribing:**

To facilitate this process, the authors describe the requisite knowledge and skills to support the seven deprescribing competencies and indicate how these map to the ten Canadian RCPSC prescribing competencies (Table 1).

**Likely gaps:** When the seven deprescribing competencies are mapped to the ten RCPSC prescribing competencies, some deprescribing competencies may be unique, such as deprescribing competency #3. For the remaining six competencies, deprescribing knowledge and skills may be partially or fully embedded within the prescribing curriculum. A review of teaching activities and curriculum subject matter can identify overlap and gaps, highlighting opportunities to include a deprescribing lens within the program.

**Suggested process steps for mapping**

To operationalize this approach, the curriculum committee can map deprescribing competencies to each prescribing competency and identify the knowledge and skills that are not included in the prescribing competencies, which can then be considered for addition to the curriculum. Below, we provide a table that can be used to identify where and how RCPSC competencies for *prescribing* and corresponding *deprescribing* competencies are taught within the curriculum and where gaps may exist.

**An option for mapping prescribing and deprescribing competencies, knowledge, and skills**

| **RCPSC Prescribing competency** | **Relevant Deprescribing competency** | **Examples where relevant knowledge and skills are taught within the curriculum** | **Examples where knowledge and skills related to the competency are assessed** | **Are knowledge and skills for deprescribing taught at an appropriate level for learners?** | **Gaps or opportunities** |
| --- | --- | --- | --- | --- | --- |
| 1.Perform a comprehensive assessment of the patient to identify a therapeutic target | 1.Conduct a comprehensive patient medication history |  |  |  |  |
| 2.Consider optimal pharmacological and nonpharmacological options | 2.Interpret relevant information in the context of desired therapeutic outcomes and goals of care  4.Assess deprescribing potential of each medication by weighing benefits and harms |  |  |  |  |
| 3.Prescribe medications appropriate to the patient’s diagnoses, considering cost and risk of benefit and harm. | 2.Interpret relevant information in the context of desired therapeutic outcomes and goals of care  4.Assess deprescribing potential of each medication by weighing benefits and harms |  |  |  |  |
| 4.Provide medication-relevant information ensuring patient/family understanding and ability to access | 5.Decide whether deprescribing a medication is appropriate using shared decision-making |  |  |  |  |
| 5.Reach a shared decision on medication use and monitoring with the patient and/or family | 2.Interpret relevant information in the context of desired therapeutic outcomes and goals of care  5.Decide whether deprescribing a medication is appropriate using shared decision-making |  |  |  |  |
| 6.Monitor and review the patient’s medications and adherence at each encounter, aiming to optimize the regimen | 6.Design, document, and share a deprescribing and monitoring plan  7.Monitor patient progress and provide support |  |  |  |  |
| 7.Prescribe carefully with attention to medication safety | 6.Design, document, and share a deprescribing and monitoring plan |  |  |  |  |
| 8.Prescribe responsibly and ethically within legal and regulatory framework | 6.Design, document, and share a deprescribing and monitoring plan |  |  |  |  |
| 9.Critically assess and improve prescribing |  |  |  |  |  |
| 10.Demonstrate leadership by fostering high quality medication management across the continuum of care |  |  |  |  |  |
|  | 3. Identify medications that are no longer necessary, may have more harm than benefit, or are otherwise potentially inappropriate |  |  |  |  |

Using the table above, considering the first RCPSC prescribing competency “Perform a comprehensive assessment of the patient to identify a therapeutic target” and its’ eight milestones reveals two mentions of medications (Takes an appropriate medical, social and medication history including allergies and intolerances; Reviews adherence to and effectiveness of current medications). A review of current curriculum to determine where and how knowledge and skills related to this competency are taught and assessed will identify opportunities to incorporate content from the knowledge and skills required for the corresponding deprescribing competency “Conduct a comprehensive patient medication history” as outlined in Table 1. For example, there may be opportunities to ensure learners understand how to conduct a Best Possible Medication History (BPMH) and have knowledge of available tools, while considering the patient’s values, beliefs, experiences and expectations related to medications.

Additionally, within Table 1, the level of learning required with respect to the list of knowledge and skills that support the corresponding competency should be determined for each type of health care professional using the Miller’s pyramid for assessing clinical competence as a guide [4]. For example, many health professionals can effectively undertake a BPMH; a medical program should teach medical students how to work with a team for this purpose and rely on their BPMH.

For Deprescribing Competency #3, where there is no corresponding RCPSC competency, one could also use a similar approach to consider the details outlined in Table 1 and assess where and how knowledge and skills related to this competency are already taught and assessed within the curriculum, then identify gaps.

**More examples and suggestions**

Ideally, prescribing and deprescribing competencies could be incorporated within a pharmacology block. However, if the curriculum does not contain a specific pharmacology block, there may be other opportunities to address polypharmacy, such as in the fields of geriatric medicine and palliative care. In addition, specific therapeutic modules can include a discussion about the importance of adjusting clinical practice guidelines to consider their applicability to older adults. For example, when discussing the treatment of diabetes or depression, review opportunities for deprescribing based on available evidence, particularly when individuals are frail, when there is evidence for increased sensitivity or adverse effects (e.g. hypoglycemia, falls, diarrhea, tremor) or when medications may no longer be needed. Here are a few more ideas for incorporating deprescribing competencies within specific courses or modules:

- Geriatrics: deprescribing competencies can be formalized within education sessions on polypharmacy or care of the patients with multimorbidity or frailty.
- Palliative Care: deprescribing competencies can be formalized within a review of potentially inappropriate medicines (unnecessary or unwanted) and this can be linked to specific randomised trials showing improved quality of life (the key outcome for palliative care).
- Diabetes: review appropriate blood glucose targets in older adults and consequences of ‘tight’ control, potential adverse effects of classes of anti-diabetic medications in older adults.
- Depression: review the evidence for efficacy, usual intended course duration, and plan for deprescribing when initiating prescribing, adverse effects of antidepressants, particularly in older adults, and provide support for management of adverse drug withdrawal effects.
- Cardiovascular risk: consider how to adjust targets for care, review treatment in the face of shifting patient priorities and evidence of benefit with age (e.g. beta blockers, statins), as well as shifts in risk benefit analysis in making decisions about reducing doses or stopping medications.

References

1. Frank JR, Snell L, Sherbino J, editors. CanMEDS 2015 Physician Competency Framework. Ottawa: Royal College of Physicians and Surgeons of Canada; 2015.
2. CanMEDS: Better Standards, Better Physicians, Better Care [Internet]*.* The Royal College of Physicians and Surgeons of Canada; [cited 2022 Nov. 15] <https://www.royalcollege.ca/rcsite/canmeds/canmeds-framework-e>
3. Brien S, Chan MK, Grill A, Holbrook A, Landiault A, Matlow, A. The Prescribing Safely Canada Physician Prescribing Competencies. Ottawa: The Royal College of Physicians and Surgeons of Canada; 2018. <https://rcportal.royalcollege.ca/mssites/prescribingsafely/EN/PSCCOMP2018.pdf>
4. Miller GE. The assessment of clinical skills/competence/performance. Acad Med. 1990;65:S63-S67. <https://doi.org/10.1097/00001888-199009000-00045>
